# Potentiation of ABCA3 lipid transport function by ivacaftor and genistein

**DOI:** 10.1111/jcmm.14397

**Published:** 2019-06-18

**Authors:** Susanna Kinting, Yang Li, Maria Forstner, Florent Delhommel, Michael Sattler, Matthias Griese

**Affiliations:** ^1^ Department of Pediatrics, Dr. von Hauner Children's Hospital University Hospital, LMU Munich Munich Germany; ^2^ Member of the German Center for Lung Research (DZL) Munich Germany; ^3^ Institute of Structural Biology Helmholtz Zentrum München Neuherberg Germany; ^4^ Center for Integrated Protein Science Munich at Department Chemie Technical University of Munich Garching Germany

**Keywords:** ABCA3, CFTR potentiators, genistein, interstitial lung disease, ivacaftor

## Abstract

ABCA3 is a phospholipid transporter implicated in pulmonary surfactant homoeostasis and localized at the limiting membrane of lamellar bodies, the storage compartment for surfactant in alveolar type II cells. Mutations in ABCA3 display a common genetic cause for diseases caused by surfactant deficiency like respiratory distress in neonates and interstitial lung disease in children and adults, for which currently no causal therapy exists. In this study, we investigated the effects of ivacaftor and genistein, two potentiators of the cystic fibrosis transmembrane conductance regulator (CFTR), on ABCA3‐specific lipid transport function. Wild‐type (WT) and functional ABCA3 mutations N568D, F629L, G667R, T1114M and L1580P were stably expressed in A549 cells. Three‐dimensional modelling predicted functional impairment for all five mutants that was confirmed by in vitro experiments (all <14% of WT functional activity). Treatment with potentiators rescued the mutants N568D (up to 114% of WT), F629L (up to 47% of WT), and G667R (up to 60% of WT), the latter variation needing higher concentrations of genistein, showing reduced affinity of the potentiator to the mutant protein. Our results present a first proof that functional ABCA3 mutations are rescued by CFTR potentiators, making them a potential therapeutical option for patients suffering from surfactant deficiency due to ABCA3 mutations.

## 1 INTRODUCTION

Pulmonary surfactant is a lipoprotein complex that lines the alveolar spaces and is synthesized, stored and secreted by alveolar type II (ATII) cells. Surfactant is crucial for normal breathing, its main function is to lower the surface tension at the air‐liquid interface to prevent end‐expiratory collapse of alveolar units.^1^, ^2^, ^3^, ^4^ The storage compartments for surfactant are the lysosome‐derived lamellar bodies (LBs). Adenosine triphosphate (ATP)‐binding cassette subfamily A member 3 (ABCA3), a lipid transporter involved in surfactant homoeostasis, is localized at the outer membrane of lamellar bodies.^5^, ^6^, ^7^, ^8^ Like all ABC transporters it is composed of two transmembrane domains (TMDs) that form a pore and two nucleotide‐binding domains (NBDs) that bind and hydrolyse ATP to generate the energy to transport surfactant lipids into the lumen of LBs.^9^, ^10^

Phosphatidylcholine (PC) is the most abundant lipid species in human pulmonary surfactant^11^ and was shown to be transported by ABCA3.^6^ We recently established a functional assay to quantify the lipid transport function of ABCA3 by assessing the fluorescence intensity of TopFluor‐labeled PC (TopF‐PC) inside ABCA3‐positive vesicles that resemble LBs in A549 cells.^12^ The overall transport activity, expressed as the fluorescence intensity per vesicle in all measured vesicles is thereby composed of three different parameters: the volume of the vesicles, the portion of filled vesicles and the fluorescence intensity in filled vesicles.

Mutations in ABCA3 lead to surfactant deficiency and pulmonary diseases like fatal respiratory distress in newborns or chronic interstitial lung disease in children (chILD) and adults.^13^, ^14^ To date, no causal therapies exist to treat patients suffering from lung diseases due to ABCA3 mutations. It is therefore a major task to identify pharmacological modulators for ABCA3 that would allow to treat those diseases.

In cystic fibrosis, a pulmonary disease caused by mutations in the ABC transporter cystic fibrosis transmembrane conductance regulator (CFTR, ABCC7),^15^, ^16^ compounds have successfully been developed, which partially or completely correct the molecular defect in a mutation‐specific manner. Misfolding mutations, like the most frequent variation F508del, that lead to impaired processing and trafficking through the cell due to ER retention can be targeted by so called correctors that increase the delivery of CFTR to the cell surface.^17^, ^18^, ^19^, ^20^ Functional mutations, like the third‐most frequent CFTR variation G551D, that display impaired function but correct processing and localization, can be rescued by potentiators.^17^, ^20^, ^21^, ^22^ CFTR potentiators ivacaftor (IVA) and genistein (GEN) lead to an increase in CFTR transport activity at the cell surface by enhancing its open probability (P_0_).

**Figure 1:**
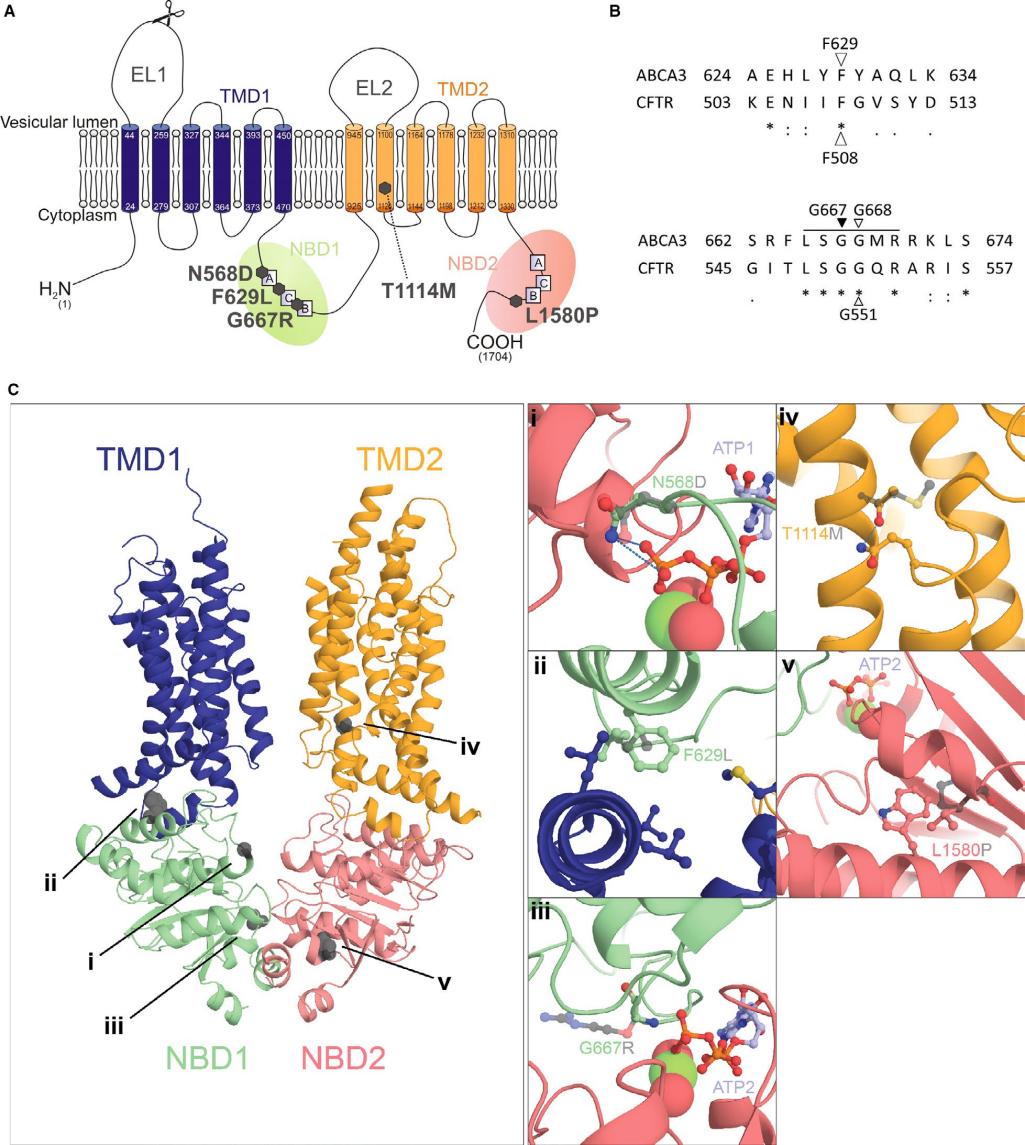
Localization and molecular consequences of five functional ABCA3 mutations. A, Two‐dimensional (2D) topology model of ABCA3 with marked positions of the five analysed functional mutations. Scissors mark cleavage site for processing of the 190 to 170 kDa form. EL: external loop, A: Walker A motif, B: Walker B motif, C: C motif. B, Sequence alignment between ABCA3 and cystic fibrosis transmembrane conductance regulator (CFTR) to identify amino acids homologous to F508 and G551 in CFTR. Variations F629L and G667R were identified using the Exome Aggregation Consortium (ExAc) Browser,^27^ with G667 corresponding to G550 in CFTR since no mutation was listed for position G668 in ABCA3. A solid line indicates the conserved ABC signature motif (LSGGQ). C, Full‐length 3D model of ABCA3 with detailed pictures of location and consequences of the five functional ABCA3 mutations (i) N568D, (ii) F629L, (iii) G667R, (iv) T1114M, (v) L1580P. Mutated residues are represented as grey spheres in the full‐length model. In the detailed pictures, the side chains of the wild‐type residues are represented in full sticks and the substituting residues are shown in transparent grey sticks at each mutated position. Other residues of interest and ATP are also represented in stick when required; likely hydrogen bonds of interest are represented as blue dotted lines. ATP: adenosine triphosphate; NBD: nucleotide‐binding site; TMD: transmembrane domain

For ABCA3 we showed that a treatment with correctors rescued processing, trafficking, localization and function of misfolding mutations.^23^ Since a lot of ABCA3 mutations are classified as functional mutations, the main goal of this study was to evaluate the effect of potentiators on the lipid transport function of those mutations. Therefore we analysed three well‐described functional ABCA3 mutants, namely N568D in NBD1, T1114M in TMD2 and L1580P in NBD2.^24^, ^25^, ^26^ We additionally analysed F629L and G667R variations, which were homologous to positions F508 and G550 in CFTR respectively (Figure 1A and B). G667R is located in the NBD1 conserved ABC signature motif, similarly affected in G551D in CFTR. It was selected because a rare variant has been described in humans in this position.^27^

All five mutants showed severely impaired lipid transport function that was rescued by treatment with the CFTR potentiators ivacaftor (IVA) and genistein (GEN) in mutants N568D, F629L and G667R. The results presented here might pave the way for mutation‐group specific treatment of pulmonary diseases caused by ABCA3 mutations.

## 2 MATERIALS AND METHODS

### 2.1 Molecular modelling

Structural models of wild‐type and mutant ABCA3 were build using phyre2 protein modelling webserver.^28^ The model of ABCA3 full‐length in its unbound conformation was build based on the 4.1Å electron microscopy structure of human ABCA1 (pdbid: 5XJY).^29^ Solely the NBD1‐2 and the TMD1‐2 were kept in the final model. These regions have an identity of 46% with the template, ensuring an accurate prediction of ABCA3 structure. The model of the NBD1‐NBD2‐ATP was modelled based on the 3.25Å crystallography structure of bacterial MacB dimer bound to ATP (pdbid: 5LJ7).^30^ The NBD1 and NBD2 of ABCA3 have identities of 30% and 22% respectively to the template NBD domain of MacB. Mutations in the TMD (T1114M) or of the TMD/NBD interface (F629L) were modelled on full‐length ABCA3 while mutations close to the ATP binding sites (N568D, G667R and L1580P) were modelled on the NBD1‐NBD2‐ATP assembly.

### 2.2 Sequence analysis

Protein sequence alignment between ABCA3 and CFTR was performed with the UniProt sequence alignment online tool.^31^ F629 and G668 were identified to correspond to F508 and G551 in CFTR (Figure 1B). Variations F629L and G667R were identified using the Exome Aggregation Consortium (ExAc) Browser,^27^ with G667 corresponding to G550 in CFTR since no mutation was listed for position G668 in ABCA3.

Conservation analysis was performed with ConSurf Server.^32^ Initial sequence selection was performed on the Uniref90 database using an E‐value threshold of 0.0001. Within this pool, the best 137 ABCA3 sequences were manually selected. Final alignment was performed with the MAFFT‐L‐INS‐i method and scoring was calculated using the Bayesian method.

### 2.3 Potentiators

Ivacaftor (VX‐770, IVA) was purchased from Sellekchem (Munich, Germany). Genistein (GEN) was purchased from Sigma Aldrich (Taufkirchen, Germany). Both substances were dissolved in dimethyl sulphoxide (DMSO, Sigma).

### 2.4 Cell culture

A549 cells were obtained from the German Collection of Microorganisms and Cell Cultures (DSMZ, Braunschweig, Germany) and cultured in RPMI 1640 medium (Life technologies, Darmstadt, Germany) supplemented with 10% foetal bovine serum (FBS, Sigma) at 37°C and 5% CO_2_.

### 2.5 Plasmids

A pT2/HB transposon vector (Addgene, Cambridge, plasmid#26557) was generated, containing hABCA3 cDNA (NM_001089) with corresponding CMV promoter elements fused to a C‐terminal HA‐tag and puromycin resistance gene, as described before.^33^ Single point mutations p.N568D (c.1702 A > G), p.F629L (c.1887 C > G), p.G667R (c.1999 G > A), p.T1114M (c.3341 C > T), and p.L1580P (c.4739 T > C) were introduced into the vector using the Q5® site‐directed mutagenesis kit (NEB, Massachusetts, United States). Primer sequences are given in the Supporting Information Materials and Methods Section.

### 2.6 Transfection and generation of stable cell clones

Transfection of A549 cells using the sleeping beauty transposon system^34^ and generation of stable cell clones were performed as described before.^33^

### 2.7 Protein isolation and Western blotting

A549 cells were lysed in radioimmunoprecipitation assay (RIPA) buffer [0.15 mol/L sodium chloride, 1% Triton‐X 100, 0.5% sodium deoxycholate, 0.1% sodium dodecylsulfate, 5 mmol/L ethylene diamine tetraacetic acid (EDTA), 50 mmol/L Tris (pH 8)] (Sigma, EDTA from GE Healthcare, Buckinghamshire, UK, Tris from Merck Millipore, Darmstadt, Germany), supplemented with complete protease inhibitor (Roche, Mannheim, Germany). Protein concentrations were measured using the Pierce BCA protein assay (Thermo Fisher Scientific, Waltham, Massachusetts, USA) and 15 µg protein was separated on NuPage Mini 3‐8% Tris‐Acetate gels (Invitrogen, Waltham, Massachusetts, USA) and subsequently transferred to a polyvinylidene fluoride (PVDF) membrane (Merck Millipore). For probing of ABCA3‐HA, rat anti‐HA monoclonal antibody (Roche) and rabbit anti‐rat IgG (H + L) HRP secondary antibody (Southern Biotechs, Birmingham, AL) were used. β‐Actin (Santa Cruz, Dallas, TX) probing served as a loading control. SuperSignal® West Femto Maximum Sensitivity Substrate (Thermo Fisher Scientific) was used for detection. Densitometric analysis was performed with Image J software.

### 2.8 Immunofluorescence staining and confocal microscopy

For immunofluorescent stainings, cells were seeded in ibiTreat slides (ibidi, Martinsried, Germany). Cells were fixed with 4% paraformaldehyde (Merck Millipore) and permeabilized with 0.5% TritonX‐100 (Sigma). Cells were incubated with blocking solution [3% bovine serum albumin (BSA, Sigma) and 10% FBS in PBS] to block unspecific binding sites. ABCA3‐HA protein and CD63 were probed with anti‐HA (Sigma) and anti‐CD63 antibody (abcam, Cambridge, UK), and according AlexaFluor secondary antibodies (life technologies). Nuclei were stained by incubation with 0.1 µg/ml 4′,6‐diamidin‐2‐phenylindol (DAPI, life technologies). Subsequently, cells were covered in mounting medium [90% glycerin in PBS and 2% 1,4‐diazabicyclo[2.2.2]octane (DABCO, Sigma)] and images were acquired using a ZEISS LSM 800 with ZEN 2 blue edition software.

### 2.9 TopFluor‐PC transport quantification

Surfactant‐like liposomes were prepared and transport of TopFluor‐labeled phosphatidylcholine (TopF‐PC) into HA‐positive vesicles was quantified as described before.^12^ In short, TopF‐PC containing liposomes (1:20 diluted in OptiMEM, Thermo Fisher Scientific) were offered to the cells expressing WT or mutant ABCA3‐HA for 30 minutes at 4°C. After two hours at 37°C, cells were treated with potentiators or DMSO as a vehicle control for 24 hours. Then cells were covered with 5% BSA (in PBS) for 30 minutes at 4°C for removal of residual labelled lipids adherent to the cell membrane. Cells were fixed, permeabilized with saponin (Carl Roth GmBH, Karlsruhe, Germany) and stained for HA‐tag. Microscopy, fluorescence analysis and quantification of vesicle volume and percentage of filled vesicles were performed as described previously^12^ using a confocal laser‐scanning microscope (LSM 800, ZEISS with ZEN 2 blue edition software) and the modified Fiji (Image J) plugin “Particle_in_Cell‐3D”.^35^

### 2.10 Statistical analysis

Data are shown as means ± SEM. Statistical significance among means was calculated using one‐way ANOVA with Dunnet's post hoc test to compare to the WT or DMSO vehicle‐treated control. *P* < 0.05 was considered significant.

## 3 RESULTS

### 3.1 ABCA3 mutations are predicted to be functional mutations according to an ABCA3 3D model

Three‐dimensional structure modelling was performed and molecular consequences of all five mutations were consistently predicted to impair function of the ABCA3 lipid transport activity (Figure 1). These observations are indicated for each mutation below.

The residue N568 is located in the conserved Walker A motif. Its side chain is directly involved in the binding of the third phosphate group of ATP. The loss of the side chain amine induced by the N568D mutation likely prevents this interaction and reduces or completely prevents ATP binding to ABCA3 (Figure 1C,i).

Residue F629, homologous to F508 in CFTR (Figure 1B), is very well conserved and is located in the NBD1 at a hydrophobic pocket, making interface with the transverse helices of TMD1. Substitution of a phenylalanine by a leucine prevents some hydrophobic interactions and may hinder the allosteric transmission of conformational changes to the TMD following ATP hydrolysis (Figure 1C,ii).

G667R is located in the conserved ABC signature motif (LSGGQ) (Figure 1B) implicated in ATP binding at the NDB1/NBD2 interface. In the ATP‐bound form G667 is in close proximity to phosphate groups 2 and 3 of the ATP molecule. A substitution to an arginine adds a large side chain that cannot be accommodated and prevents ATP binding (Figure 1C,iii).

Residue T1114 is located in the second helix of the TMD2, far from the NBDs (Figure 1C,iv). It likely forms a hydrogen bond with the conserved residue Q929 in the loop following the first transverse helix of the TMD2. Its mutation to a methionine precludes this hydrogen bonding and might prevent conformational changes of the TMD2 happening upon ATP hydrolysis in the NBDs and thereby reduce the transport activity of the protein.

Residue L1580 is located in a highly conserved helix following the H‐loop. Its mutation to a proline, incompatible with helical secondary structure, has likely a strong impact on the conformation of this region and of the close H‐loop that interacts with ATP and the NBD1 (Figure 1C,v).

In summary, based on these data from 3D modelling, we expected functional impairment of all five mutants, making them possible targets for treatment with potentiators to rescue the functional defect.

### 3.2 Functional ABCA3 mutants display correct subcellular localization and processing but impaired lipid transport function in A549 cells

**Figure 2:**
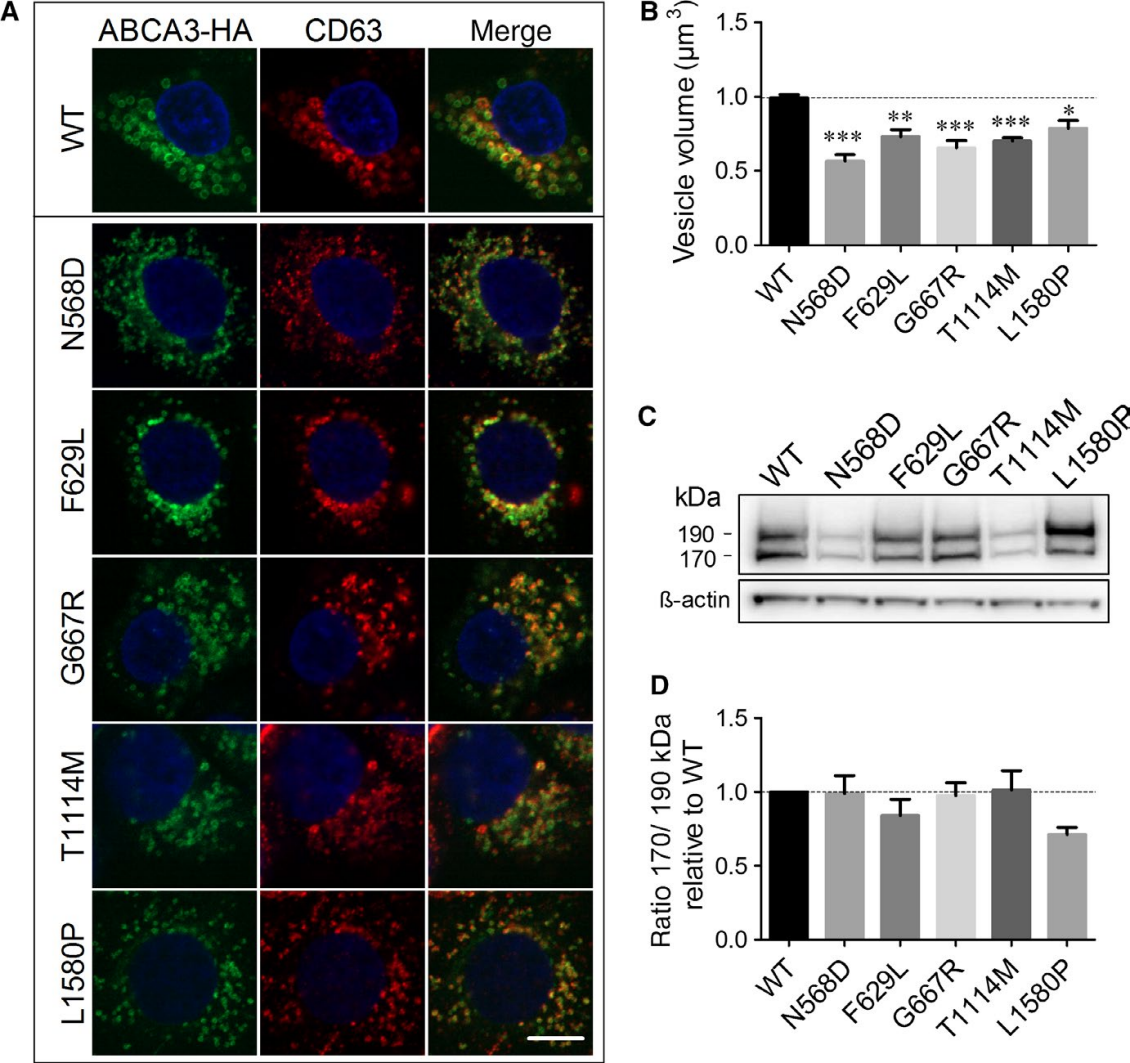
Subcellular localization and protein processing is not affected by functional mutations. A, Subcellular localization of ABCA3 wild‐type (WT) and mutants shown by immunofluorescence and confocal microscopy. A549 cells stably expressing ABCA3 WT or mutants were fixed, permeabilized and stained for ABCA3‐HA and lysosomal marker CD63. All proteins are localized in vesicular structures resembling lamellar bodies, co‐localizing with CD63. Scale bar represents 10 µm. B, Volume of ABCA3‐HA‐positive vesicles in A549 cells stably expressing WT and mutant protein. All analysed mutations led to significantly smaller vesicles compared to WT ABCA3 in A549 cells. Results are taken from the functional assay shown in Fig. 3D and are given as means ± SEM of three independent experiments. *P < 0.05 **P < 0.01 ***P < 0.001 compared to WT. C, Western blot analysis of WT and mutant ABCA3. Molecular masses are indicated on the left, β‐actin served as a loading control. D, Quantification of the Western blot shown in (C). Densitometric quantification of protein amount was performed with ImageJ. The ratio of 170/190 kDa processing form serves as a marker for correct processing. Mutants do not show different ratios from WT, indicating correct trafficking and processing in the cell. Results are means ± SEM of five independent Western blots

WT ABCA3‐HA and all five mutant proteins were stably expressed in A549 cells and showed vesicular structures that resemble LBs and co‐localized with the lysosomal marker CD63, demonstrating their correct protein localization (Figure 2A). However, the vesicles formed by all mutant proteins were significantly smaller than those in WT‐ABCA3 expressing cells (Figure 2B).

N‐terminal cleavage of ABCA3 in post‐Golgi compartments resulting in the presence of two products of about 190 and 170 kDa,^36^, ^37^ serves as a marker for correct protein trafficking.^23^, ^37^, ^38^ All five mutant proteins showed both processing products in Western blots (Figure 2C) with a ratio of 170 to 190 kDa form not significantly different from the WT protein, indicating correct processing and trafficking through the cell (Figure 2D).

**Figure 3:**
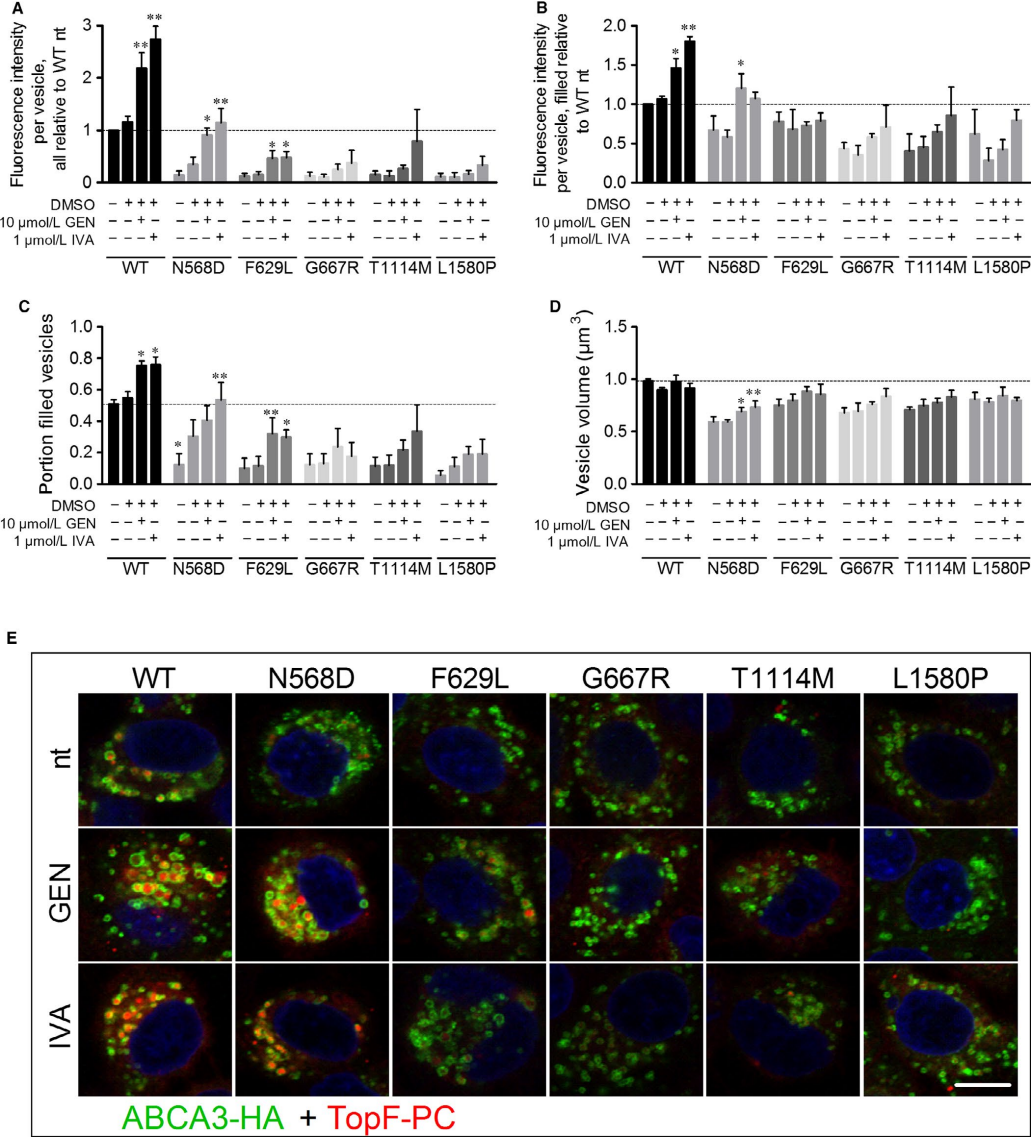
Transport of TopF‐labeled phosphatidylcholine (TopF‐PC) is increased by potentiators ivacaftor and genistein. A549 cells expressing wild‐type (WT) or mutant ABCA3 were incubated with liposomes containing TopF‐PC and treated with potentiators for 24 h. After fixation and staining for ABCA3‐HA, confocal microscopy pictures were obtained to measure: A, TopF‐PC fluorescence intensity per vesicles in all analysed ABCA3‐HA‐positive vesicles relative to WT nt, B, TopF‐PC fluorescence intensity in only TopF‐PC‐filled ABCA3‐HA‐positive vesicles relative to WT nt, C, Portion of TopF‐PC‐filled vesicles and D, Volume of all analysed ABCA3‐HA‐positive vesicles. E, Representative pictures of the experiment. Scale bar represents 10 µm. Pseudo colours were used to stay consistent with former experiments. Results are means ± SEM of three independent experiments. *P < 0.05 **P < 0.01 compared to dimethyl sulphoxide (DMSO) vehicle controls. nt: no treatment; GEN: genistein, IVA: ivacaftor; TopF‐PC: TopFluor‐labeled phosphatidylcholine

Despite normal processing and localization, all five mutants exhibited a strong decrease in lipid transport activity compared to WT as predicted from the 3D model (Figure 3A and E, no treatment). Volume of the analysed vesicles, the portion of filled vesicles and the fluorescence intensity in filled vesicles were diminished for all mutants compared to WT, resulting in a transport activity of 14% of WT in N568D and T1114M mutants, 12% activity of WT in F629L and G667R mutants and 10% of WT lipid transport activity in L1580P mutant (Figure 3A‐D, no treatment).

### 3.3 CFTR potentiators ivacaftor and genistein improve WT ABCA3 transport function and rescue functional defects of some ABCA3 mutants

In WT ABCA3‐HA expressing cells, treatment with 1 µmol/L ivacaftor or 10 µmol/L genistein led to a 2.7‐ and twofold increase of lipid transport activity, respectively, resulting from an increase in the portion of filled vesicles and the fluorescence intensity in filled vesicles (Figure 3).

In N568D expressing cells, potentiator treatment led to a drastic elevation of lipid transport activity from 14% of WT activity to 114% in presence of ivacaftor and 90% with genistein, resulting from an increase in vesicle volume, portion of filled vesicles and fluorescence intensity in filled vesicles to a WT level (Figure 3).

Lipid transport activity of F629L mutant was increased to 47% of WT activity by ivacaftor and 46% by genistein treatment, due to a significant increase of the portion of filled vesicles (Figure 3A and C).

For all other mutant proteins, a slight yet not significant increase of lipid transport activity upon potentiator treatment was detected.

Potentiators did not influence the processing of ABCA3 assessed by Western blotting (Figure S1). To further address specificity of their effects, potentiators were also tested in A549 cells expressing misfolding mutants Q215K and K1388N. Neither their processing and thus nor their function was affected (Figure S2), confirming exclusive effects of potentiators on functional mutations.

To further rule out a potential influence of the lower protein expression of N568D ABCA3‐HA on the results obtained, we additionally tested another cell clone (N568D‐2) with a higher ABCA3‐HA expression than in WT ABCA3‐HA cells and confirmed the results reported above (Figure S3).

### 3.4 G667R mutant displayed lower affinity to genistein and was rescued by increased concentrations

Genistein was shown to bind to the LSGGQ signature motif in CFTR.^39^, ^40^, ^41^ Since G667R is located in this motif in ABCA3 (Figure 1B), we suggested a decreased affinity of genistein to the mutant protein that might be overcome by higher concentrations of the potentiator.

**Figure 4:**
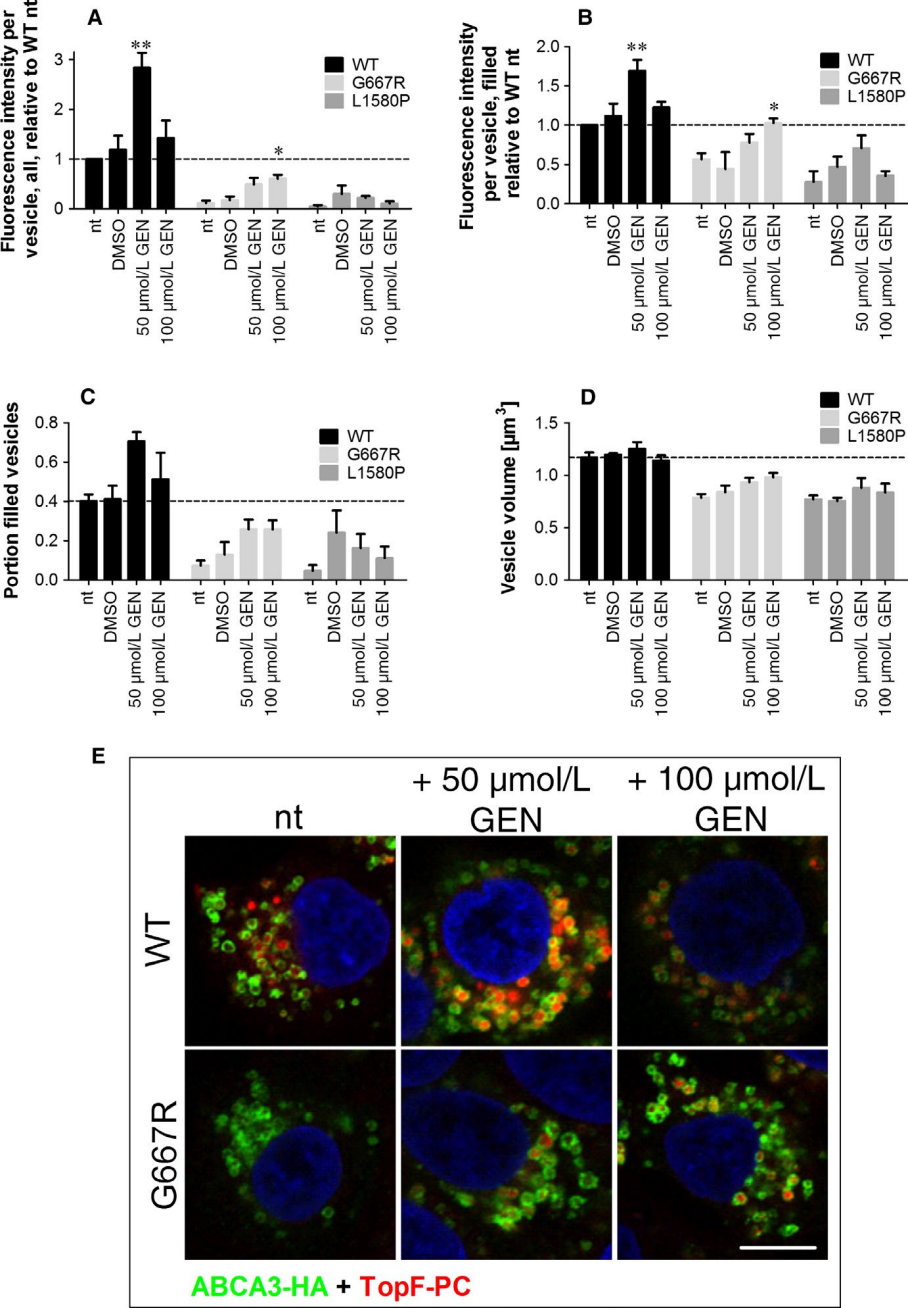
G667R ABCA3 mutant is rescued by increased concentrations of genistein. A549 cells expressing wild‐type (WT) or mutant ABCA3 were incubated with liposomes containing TopFluor‐conjugated PC (TopF‐PC) and treated with 50 or 100 μmol/L genistein (GEN) for 24 h. After fixation and staining for ABCA3‐HA, confocal microscopy pictures were obtained to measure: A, TopF‐PC fluorescence intensity per vesicles in all analysed ABCA3‐HA‐positive vesicles relative to WT nt, B, TopF‐PC fluorescence intensity in only TopF‐PC‐filled ABCA3‐HA‐positive vesicles relative to WT nt, C, Portion of TopF‐PC‐filled vesicles, and D, Volume of all analysed ABCA3‐HA‐positive vesicles. E, Representative pictures of the experiment. Scale bar represents 10 µm. Pseudo colours were used to stay consistent with former experiments. Results are means ± SEM of three independent experiments. *P < 0.05 **P < 0.01 compared to dimethyl sulphoxide (DMSO) vehicle controls. nt: no treatment; GEN: genistein, TopF‐PC: TopFluor‐labeled phosphatidylcholine

About 50 µmol/L of genistein led to a 2.8‐fold increase of lipid transport function in WT‐ABCA3 expressing cells resulting from an increase in fluorescence intensity in filled vesicles and the portion of filled vesicles, whereas 100  µmol/L genistein did not increase the lipid transport (Figure 4A and B).

In G667R expressing cells, 100 µmol/L genistein led to a significant increase in lipid transport to 60% of WT function. Assessing filled vesicles only, this concentration increased lipid transport to a WT level (Figure 4A and B) and also the portion of filled vesicles and the volume were slightly also not significantly increased (Figure 4C and D).

In L1580P ABCA3 expressing cells, no significant effect of genistein at increased concentrations was detected.

In our experimental setup, concentrations of 5 µmol/L ivacaftor reduced the viability of the cells (data not shown), so no analysis of higher concentrations of ivacaftor on lipid transport in G667R ABCA3 expressing cells was possible. Viability assays showed the toxicity of ivacaftor for the cells (Figure S4).

## 4 DISCUSSION

Functional impairment of ABCA3 due to mutations may lead to fatal or chronic disturbances of ATII cells and surfactant homoeostasis resulting in pulmonary diseases like neonatal respiratory distress syndrome and chronic interstitial lung disease. In the present study, we showed impaired phospholipid transport function of ABCA3 due to distinct disease causing functional mutations that can be rescued by the CFTR potentiators ivacaftor and genistein for mutations located in the NBD1 of the protein.

The functional defect displayed by mutants N568D and F629L was successfully rescued by 1 µmol/L ivacaftor or 10 µmol/L genistein. For mutant G667R, 100 µmol/L genistein was sufficient to yield a significant increase in lipid transport function. Like described before, mutation N568D led to a functional impairment of the ABCA3 protein with only 14% of WT transport function despite correct processing and localization.^25^ Furthermore, the two mutants F629L and G667R also showed functional impairment with 12% of WT activity but normal processing and localization and were therefore also classified as functional mutations. Ivacaftor and genistein treatment elevated transport activity of WT ABCA3 by 2.7‐ and twofold, of N568D mutant up to 114% and 90% of WT function, respectively, and 46% and 47% for F629L mutant.

Since genistein is presumably binding in the LSGGQ motif of NBD1,^41^ where the mutation G667R is located, the affinity of the potentiator to the mutant protein is likely to be lowered like shown for G551D in CFTR.^39^, ^40^ For WT ABCA3, genistein treatment exerted potentiating effects up to a concentration of 50 µmol/L and inhibitory effects at higher concentrations resulting in a bell‐shaped dose‐response relation like also shown for CFTR.^39^, ^42^, ^43^ This is explained by the assumption of two binding sites for genistein, one high‐affinity site activating the protein and a second low affinity site exerting an inhibitory effect.^41^, ^43^ In G667R ABCA3 expressing cells, on the other hand, only 100 µmol/L genistein yielded a significant increase in lipid transport activity to a level of 60% of WT function. Therefore the dose‐response curve was shifted to the right compared to WT ABCA3, indeed indicating a reduced binding of genistein. Lowered affinity of potentiators to CFTR protein harbouring the G551D mutation was also shown for various other potentiator compounds including ivacaftor.^22^, ^44^, ^45^ In our cell model, higher concentrations of ivacaftor reduced the viability of the cells and we could not evaluate their effects on ABCA3 activity. In the TopF‐PC transport assay, a concentration of 5 µmol/L ivacaftor impaired cell viability, impeding evaluation of lipid transport. In such experiments, the cells are incubated at 4°C and in serum‐reduced medium, so that treatment with ivacaftor probably adds an additional stressor to the cells. Cell type‐specific toxicity may be related to differences in cellular uptake of the drug.^46^

Impaired function of T1114M and L1580P mutants was not rescued by potentiator treatment. We recorded a lipid transport function of 14% and 10% of WT function for T1114M and L1580P as reported before.^25^, ^26^ For mutation T1114M, Matsumura *et al* assessed a rather moderate impairment of 52% of WT ATP hydrolysis function but showed a decreased lipid transport function not different from untransfected cells.^26^

The residue T1114 is located in the TMD, where it likely ensures the transmission of conformational changes triggered by NBD dimerization to the TMDs and the extracellular domain, required to translocate the substrate. Mutation of this threonine to methionine likely decouples NBD dimerization and substrate translocation, explaining the lack of effect induced by potentiators that stabilize the NBD dimer formation to enhance transport function and activity. This is further supported by the fact that ivacaftor was also ineffective to rescue the L927P CFTR mutant (T1114 is homologous to L935 in CFTR), which is also located in the eighth transmembrane helix and is implicated in conformational changes necessary to open the channel.^47^, ^48^ Furthermore, ivacaftor did not overcome impaired PC secretion activity in a TMD mutant of ABCB4.^49^

Residue L1580 is not directly located in the ATP binding site, however its mutation to a proline most likely breaks the helix, in which it is located. This will affect the upstream H‐loop, which is also implicated in NBD dimerization and ATP binding. In addition to preventing the ATP‐induced NBD dimerization, it is possible that the change in conformation might actively prevent the mutated protein to reach the active state even in presence of potentiators, explaining its non‐responsiveness even at high concentrations.

Furthermore, since ivacaftor was chemically adjusted to specifically act on CFTR^22^ it might only exert effects on regions of ABCA3 that show very high homology to CFTR, like the NBD1, which might explain exclusive effects on mutations located in this domain.

In this study, we used the A549 cell model stably expressing WT and mutant ABCA3. A limitation of this approach is the current inability to predict the effect of potentiators in patients. On the one hand, there is a lack of information on influences of the patient‐specific genetic and environmental background. On the other hand, the impact of overexpression of ABCA3 is unknown. In future studies, those limitations might be overcome by the use of patient‐specific primary cell cultures or induced pluripotent stem (iPS) cells. The optimal model would utilize patient‐derived alveolar epithelial type II cells, which are not readily available due to rarity of the patients and difficulties to access the terminal area of the lungs.

Nevertheless, the A549 model is a valuable tool to identify groups of mutations that can be targeted by the same modulator. Similar to cystic fibrosis, where in vitro studies on Fisher rat thyroid cells expressing rare CFTR mutants were sufficient for the approval of ivacaftor for 23 rare CFTR mutations without need of patient data from clinical trials.^48^, ^50^ Our functional assay using TopF‐PC reliably reproduced lipid transport and ATPase activity studies of the mutant proteins performed by Matsumura et al^25^, ^26^ (Table S1) and also replicated dose‐response relations of genistein in CFTR,^39^, ^42^, ^43^ making it suitable for high‐throughput screens to identify other substances that act as potentiators for ABCA3.

Here we showed that some functional ABCA3 mutations were rescued by the potentiators genistein and ivacaftor. This provides a proof of principle and a first step for the development of pharmacological therapies for interstitial lung diseases caused by ABCA3 mutations, for which currently no treatment is available.

## CONFLICT OF INTEREST

The authors confirm that there are no conflict of interest.

## AUTHORS CONTRIBUTION

MG, SK designed the study; SK, YL, MF performed the research; FD, MS performed the 3D modelling and analysed putative consequences of mutations; SK, MG analysed the data and wrote the manuscript. All authors read and approved the final manuscript.

## Data Availability

The data that support the findings of this study are available from the corresponding author upon reasonable request.
