# Supplementary material for: Potentiation of ABCA3 lipid transport function by ivacaftor and genistein
Source: J Cell Mol Med. 2019 Jun 18;23(8):5225–34. doi: 10.1111/jcmm.14397 (PMC6652914; doi:10.1111/jcmm.14397)
Supplement: Supplementary file 1 [file JCMM-23-5225-s001.docx]

## Supporting Information

## Supplemental material and methods

### Primer

Side directed mutagenesis was performed with the following primers (mutated nucleotides are underlined):

N568D forward: 5’-GCTGGGCCACGACGGTGCCGG-3’

N568D reverse: 5’-AGGACGGTGATCTGTCCCTCGTACAG-3’

F629L forward: 5’-ACCTTTATTTGTACGCCCAGC-3’

F629L reverse: 5’-GCTCTGCGACTGTCAAGT-3’

G667R forward: 5’-CTTCCTGAGCAGGGGCATGAG-3’

G667R reverse: 5’-CGGCTCCGTGAGTTCCAC-3’

T1114M forward: 5’-TTGGCCAGCATGTTCTCCATC-3’

T1114M reverse: 5’-GAATGCCATGGCGAAGAG-3’

L1580P forward: 5’-TGTGAGGCCCCGTGCACCCGG-3’

L1580P reverse: 5’-CTCCTCCATGCTGTGGGAGG-3’

Resulting constructs were verified via Sanger sequencing using Clone Manager Suite (Version 6.00).


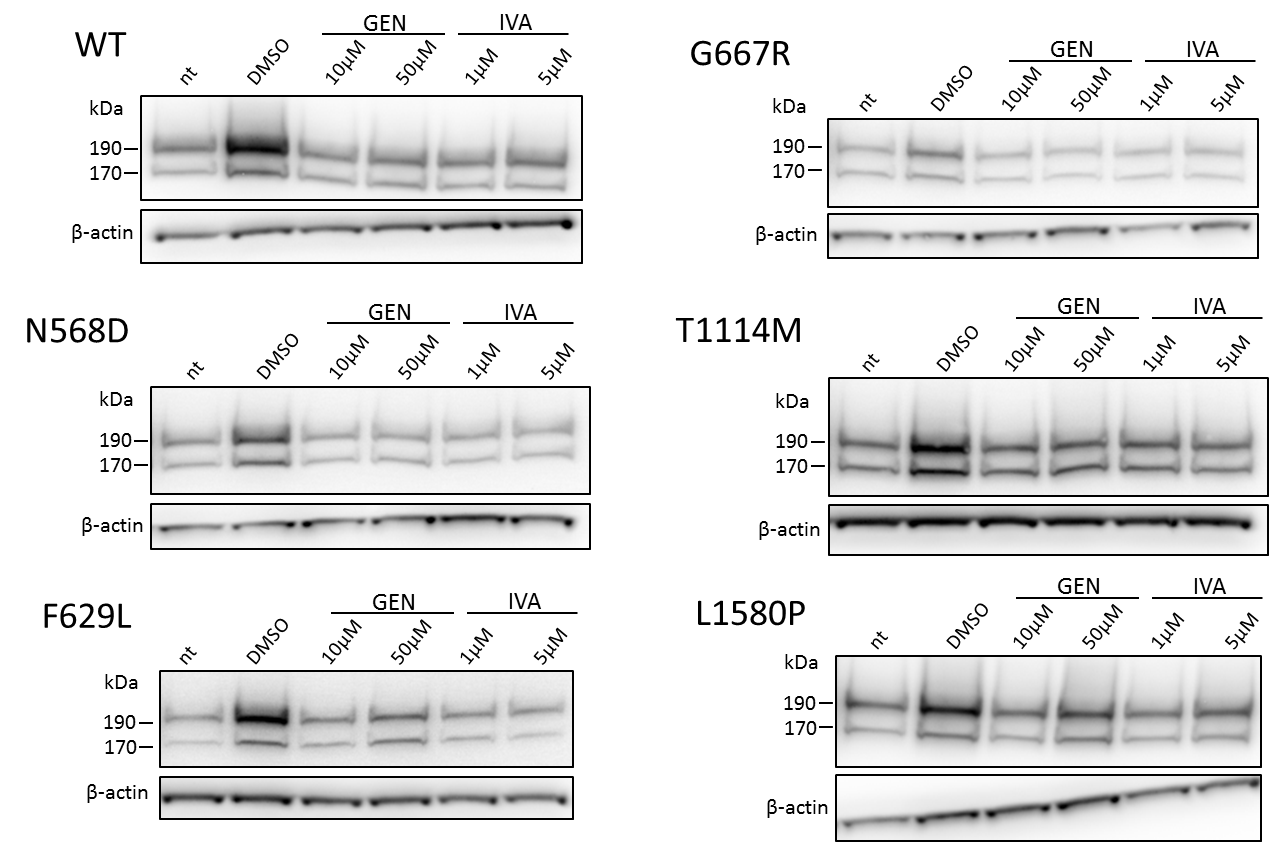


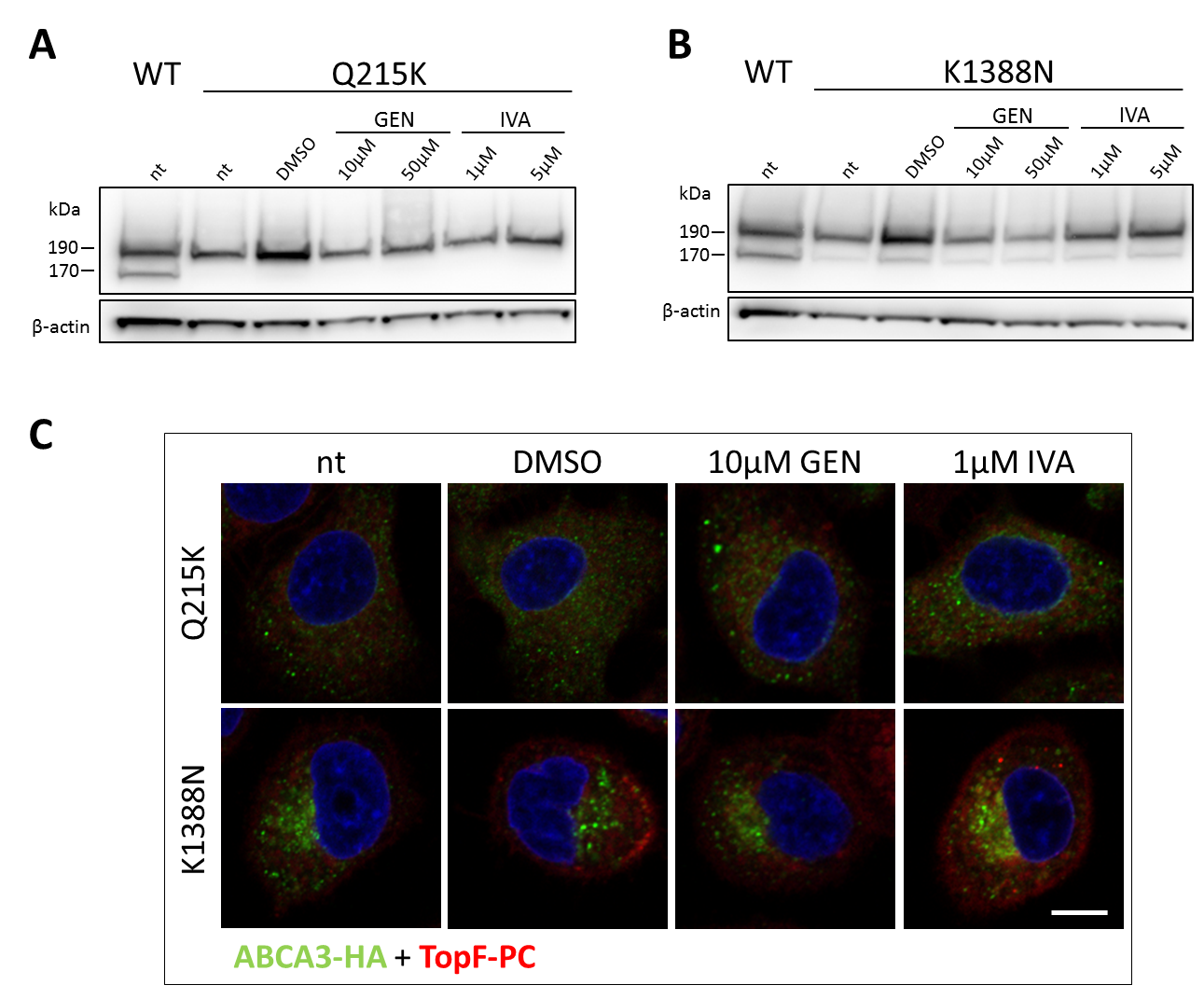


Supplemental figure S1: Potentiator treatment is not affecting protein processing.

Western blot analysis of WT ABCA3-HA and all five mutants after potentiator treatment. Treatment with potentiators genistein (GEN) and ivacaftor (IVA) had no effect on protein processing. Molecular masses are indicated on the left, β-actin served as a loading control.

Supplemental figure S2: Potentiators have no effects on misfolding mutations.

A) Western blot analysis of Q215K- and K1388N-ABCA3. Impaired processing of misfolding mutations is not affected by potentiator treatment. Molecular masses are indicated on the left, β-actin served as a loading control.

B) Representative pictures obtained by confocal microscopy. Potentiator treatment does not affect localization of Q215K- or K1388N-ABCA3-HA. Therefore no lipid transport can be assessed. Scale bar represents 10 µm.

GEN: genistein; IVA: ivacaftor


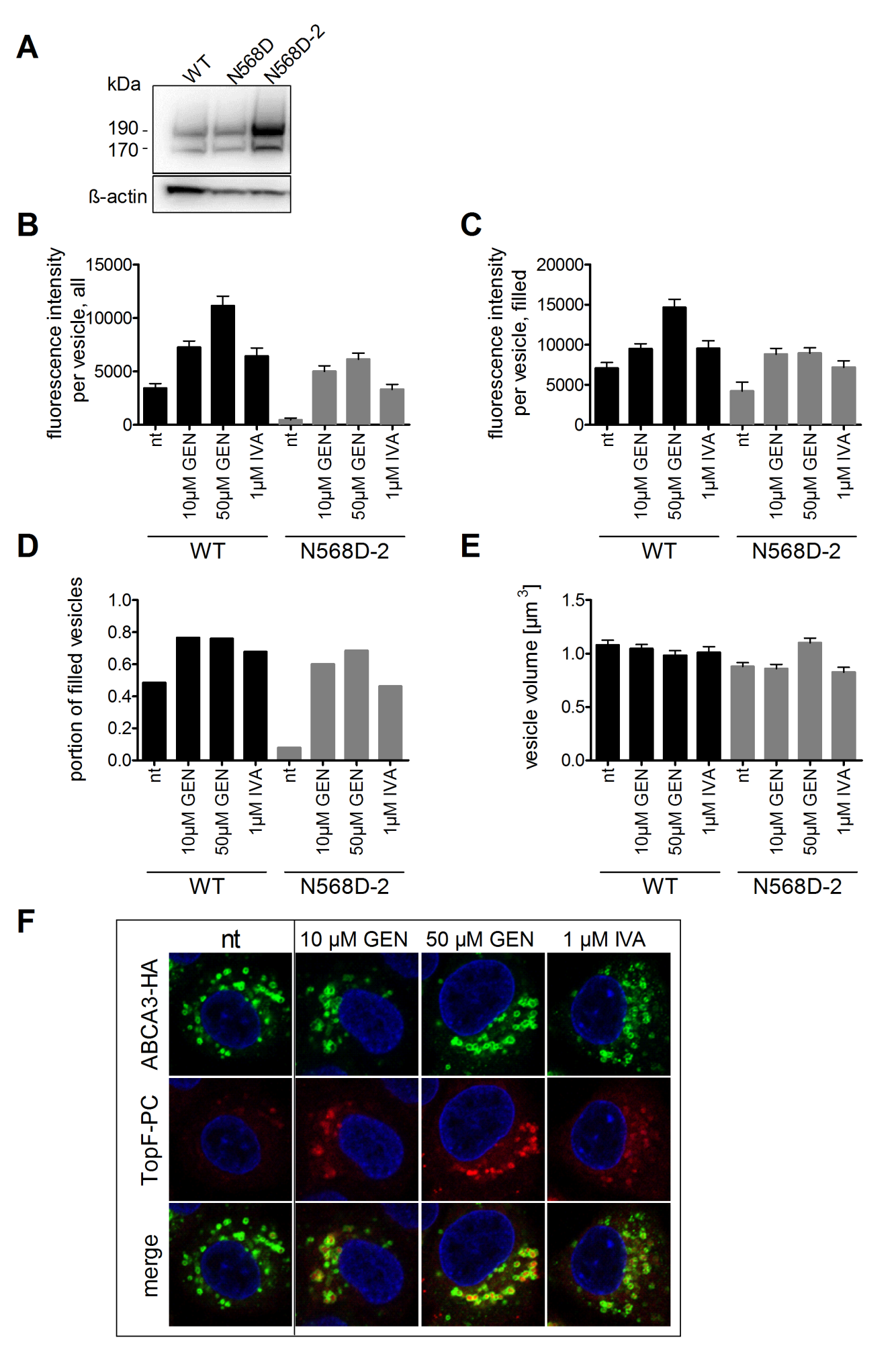


Supplemental figure S3: Transport of TopF-labeled PC in N568D-2 mutant is increased upon potentiator treatment.

A) Western blot analysis of WT ABCA3-HA and two different clones of mutant N568D ABCA3-HA. Mutant N568D-2 shows much higher protein expression than WT and N568D mutant. Molecular masses are indicated on the left, β-actin served as a loading control.

A549 cells expressing WT or mutant ABCA3 were incubated with liposomes containing TopFluor conjugated PC (TopF-PC) and treated with potentiators genistein (GEN) or ivacaftor (IVA) for 24 hours. After fixation and staining for ABCA3-HA, confocal microscopy pictures were obtained to measure

B) TopF-PC fluorescence intensity per vesicle in all analyzed ABCA3-HA positive vesicles,

C) TopF fluorescence intensity in only filled ABCA3-HA positive vesicles,

D) Portion of filled vesicles, and

E) Volume of all analyzed ABCA3-HA positive vesicles.

F) Representative pictures of the experiment showing N568D-2 ABCA3-HA mutant. Scale bar represents 10 µm. Pseudo colors were used to stay consistent with former experiments.

Results are means ± S.E.M. of 120 analyzed vesicles. nt: no treatment; GEN: genistein; IVA: ivacaftor; TopF-PC: TopFluor-labeled phosphatidylcholine


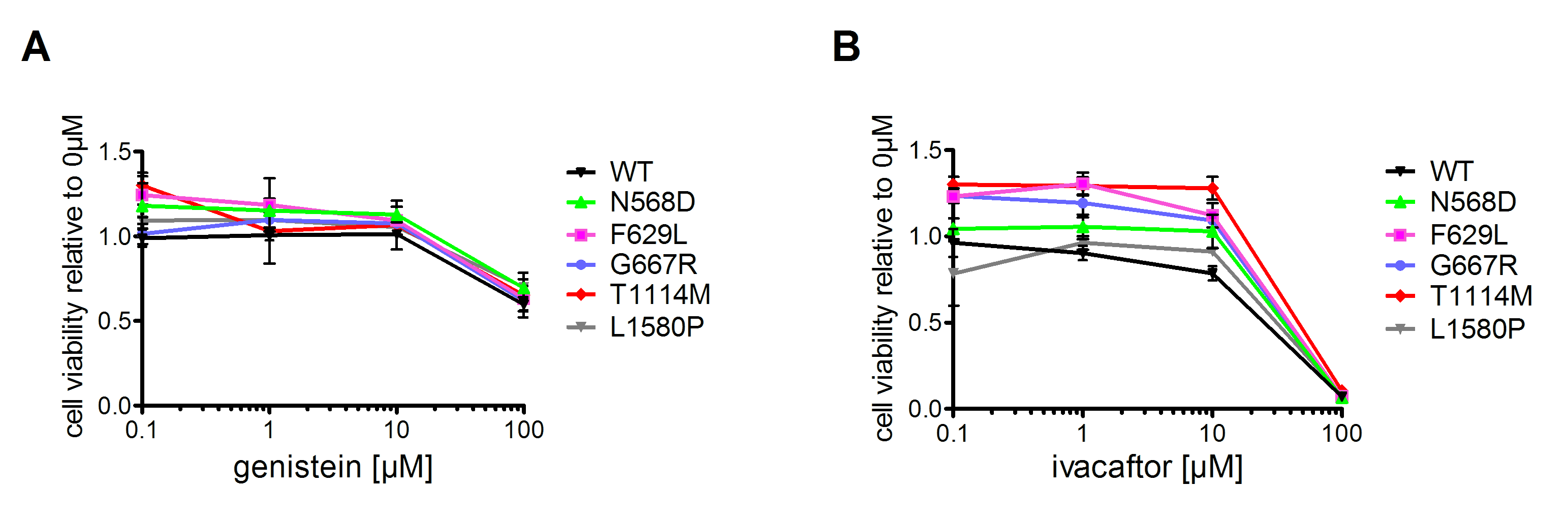


Supplemental figure S4: Cell viability upon potentiator treatment.

A549 cells stably expressing WT or mutant ABCA3-HA were treated with increasing concentrations of potentiators genistein (GEN) and ivacaftor (IVA) for 24 hours. Cell viability was assessed by quantification of the specific cleavage of yellow XTT tetrazolium salt (Sigma) to orange formazan in the presence of phenazine methosulfate (PMS, Sigma). Absorbance at 450 nm was measured using a spectrophotometer.

**Supplemental table 1: Overview of potentiator effects on WT and mutant ABCA3.**

Results of functional assays performed in this study are summarized and compared to results obtained before. nt: no treatment.

|  |  | Fluorescence intensity in all vesicles relative to WT nt | | |
| --- | --- | --- | --- | --- |
|  | ATPase activity % WT * | nt | Genistein (10µM) | Ivacaftor (1µM) |
| WT | 100 | 100 | **217 ± 30** | **272 ± 25** |
| N568D | 13 | 14 ± 8 | **90 ± 14** | **114 ± 26** |
| F629L | - | 12 ± 5 | **46 ± 15** | **47 ± 11** |
| G667R | - | 12 ± 7 | **60 ± 8^×^** | 37 ± 24 |
| T1114M | 52^†^ | 14 ± 7 | 26 ± 7 | 78 ± 61 |
| L1580P | 9 | 10 ± 7 | 16 ± 6 | 33 ± 16 |

**bold**: significant change compared to DMSO vehicle control

*: measurements by Matsumura *et al*. (1, 2)

^†^: lipid transport function not different from untransfected cells (without ABCA3)

×: treatment with 100µM genistein

**References**

1. Matsumura, Y., Ban, N., Ueda, K. and Inagaki, N. (2006) Characterization and classification of ATP-binding cassette transporter ABCA3 mutants in fatal surfactant deficiency. *J. Biol. Chem.*, **281**, 34503-34514.
2. Matsumura, Y., Ban, N. and Inagaki, N. (2008) Aberrant catalytic cycle and impaired lipid transport into intracellular vesicles in ABCA3 mutants associated with nonfatal pediatric interstitial lung disease. *Am. J. Physiol. Lung Cell Mol. Physiol.*, **295**, L698-707.
